# A longitudinal study of free leptin index in pre‐eclamptic pregnancies

**DOI:** 10.1111/jcmm.17707

**Published:** 2023-03-23

**Authors:** María Fernanda Garcés, Julieth Daniela Buell‐Acosta, Haiver Antonio Rodríguez‐Navarro, María Carolina Páez‐Leal, Luis Miguel Maldonado‐Acosta, Jhon Jairo Peralta‐Franco, Álvaro Javier Burgos‐Cardenas, Edith Ángel‐Müller, Arturo José Parada‐Baños, Mario Orlando Parra‐Pineda, Javier Eslava‐Schmalbach, Camilo Andrés Escobar‐Sarmiento, Ezequiel Lacunza, Sofia Alexandra Caminos‐Cepeda, Justo P. Castaño, Rubén Nogueiras, Carlos Dieguez, Ariel Iván Ruiz‐Parra, Jorge Eduardo Caminos

**Affiliations:** ^1^ Department of Physiology School of Medicine Universidad Nacional de Colombia Bogotá Colombia; ^2^ Department of Public Health School of Medicine Universidad Nacional de Colombia Bogotá Colombia; ^3^ Division of Endocrinology – Department of Internal Medicine School of Medicine Universidad Nacional de Colombia Bogotá Colombia; ^4^ Department of Internal Medicine School of Medicine Universidad Nacional de Colombia Bogotá Colombia; ^5^ Department of Obstetrics and Gynecology School of Medicine Universidad Nacional de Colombia Bogotá Colombia; ^6^ Department of Surgery School of Medicine Universidad Nacional de Colombia Bogotá Colombia; ^7^ Fundación Sueño Vigilia Colombiana Bogotá Colombia; ^8^ Centro de Investigaciones Inmunológicas Básicas y Aplicadas (CINIBA), Facultad de Ciencias Médicas Universidad Nacional de La Plata La Plata Argentina; ^9^ School of Medicine Universidad Pompeu Fabra Barcelona Spain; ^10^ Department of Cell Biology, Physiology, and Immunology Institute Maimonides for Biomedical Research of Cordoba, Reina Sofia University Hospital, University of Cordoba Cordoba Spain; ^11^ CIBER Fisiopatología de la Obesidad y Nutrición Instituto de Salud Carlos III Madrid Spain; ^12^ Department of Physiology (CIMUS) School of Medicine ‐ Instituto de Investigaciones Sanitarias (IDIS), Universidad de Santiago de Compostela Santiago de Compostela Spain

**Keywords:** free leptin index, pregnancy and pre‐eclampsia

## Abstract

The ratio between circulating levels of leptin and soluble leptin receptor (sOB‐R), the free leptin index (FLI), is used as a marker of leptin resistance. Therefore, the aim of our study was to investigate the FLI in mild pre‐eclamptic pregnancies in a nested case–control study within a prospective observational study. Circulating levels of leptin and sOB‐R levels rise significantly during pregnancy in healthy (*p* < 0.05) (*n* = 46) and pre‐eclamptic pregnancies (*p* < 0.05) (*n* = 20). Serum levels of leptin were significantly higher in pre‐eclamptic compared to healthy pregnancies at second and third trimesters of pregnancy (*p* < 0.05). Additionally, serum levels of sOB‐R were significantly lower in pre‐eclamptic pregnancies during the second and third trimesters of pregnancy compared to healthy pregnancies (*p* < 0.05). Moreover, we found that FLI did not vary significantly during pregnancy in healthy women (*p* > 0.05), while it increases in pre‐eclamptic pregnancies (*p* < 0.05). Indeed, FLI was significantly higher at second and third trimesters of pregnancy in pre‐eclamptic compared to healthy pregnancies (*p* < 0.05). In addition, FLI was significantly higher in the luteal phase compared with the follicular phase of the menstrual cycle in eumenorrheic women (*p* < 0.05). Receiver operating characteristic (ROC) curve analysis revealed the ability of leptin (AUC = 0.72) and FLI (AUC = 0.67) as a reliable predictor for mild pre‐eclampsia during the second trimester of pregnancy. In conclusion, our findings show that FLI were significantly increased in mild pre‐eclamptic pregnancies and allowed us to hypothesize that this rise might alter leptin bioavailability and bioactivity which might lead to the sympathetic hyperactivity and the hypertensive disorders during pregnancy.

## 1 INTRODUCTION

Leptin is an adipokine mainly synthesized and secreted by adipose tissue. Leptin plays pleiotropic roles in controlling energy metabolism homeostasis, reproductive function modulation, immune balance and bone metabolism, by binding to the cell surface receptor.^1^ Previous studies have described six leptin receptors splice variants of the class I cytokine receptor family with common ligand‐binding domains and alternative or truncate cytoplasmic domains.^1^, ^2^ Additionally, leptin may circulate free as a biologically active protein or bound to the soluble leptin receptor (sOB‐R). The sOB‐R is the main leptin‐binding protein in circulation and plays a critical role in leptin signalling pathways.^3^, ^4^, ^5^, ^6^, ^7^, ^8^, ^9^

On the other hand, circulating levels of leptin rise significantly during normal pregnancy, reaching a nadir in late pregnancy and returning to preconception levels in the postpartum.^10^ Additionally, different studies have demonstrated that chronically high levels of leptin are related to the metabolic syndrome and gestational diabetes mellitus.^7^, ^11^, ^12^, ^13^, ^14^, ^15^, ^16^ Furthermore, previous studies have shown that high levels of leptin may affect blood pressure and contribute to hypertension mediated by sympathetic nervous system activation.^17^, ^18^ It is important to highlight that the basal sympathetic nerve activity increases during normal pregnancy, therefore, any deregulation of leptin levels might play a critical role in the pathogenesis of pre‐eclampsia.^10^, ^19^, ^20^, ^21^, ^22^, ^23^ In addition, different studies have shown that gene expression of leptin is increased in pre‐eclamptic placentas and circulating levels of leptin are higher in pre‐eclamptic compared with normotensive pregnancies.^24^, ^25^, ^26^, ^27^

The ratio between circulating levels of leptin and sOB‐R, the free leptin index (FLI), has been used to assess leptin resistance.^26^, ^27^, ^28^ FLI is significantly increased in obese individuals, due to higher levels of leptin and lower levels of sOB‐R compared to healthy weight controls or obese subjects undergoing a weight reduction diet.^29^, ^30^, ^31^ In this way, FLI have been associated with chronic progressive diseases such as obesity, type 2 diabetes (T2D), reproductive diseases and metabolic‐associated fatty liver disease (MAFLD).^13^, ^32^, ^33^, ^34^, ^35^ Also, FLI has been associated with different pathological pregnancy states, including pre‐eclampsia and gestational diabetes mellitus (GDM).^7^, ^13^, ^36^, ^37^

Different cross‐sectional studies have demonstrated, during pregnancy, that FLI is significantly increased in pre‐eclamptic compared with healthy pregnancies.^36^, ^38^, ^39^ FLI studies have not been performed to describe the longitudinal profile in mild pre‐eclamptic pregnancy. Hence, this study aims to assess the FLI profile in mild pre‐eclamptic and healthy pregnancies in a case–control study nested within a longitudinal prospective cohort.

## 2 METHODS

### 2.1 Ethical considerations

This protocol study was approved by the Institutional Ethics Committee of the School of Medicine of the Universidad Nacional de Colombia (Reference Number No. 011‐165 – 18). The authors conducted this nested case–control study within a longitudinal observational prospective cohort study at the Gynaecology and Obstetrics Department of the School of Medicine – Universidad Nacional de Colombia and the tertiary referral hospital of Engativa ‐Bogotá.

### 2.2 Study design

This study was carried out in order to compare maternal FLI in healthy and pre‐eclamptic pregnancies, during each trimester of pregnancy and 3 months after childbirth. Women were recruited in the first trimester of pregnancy (11–13 weeks) and followed up to 3 months postpartum at the obstetrics and gynaecology health promotion and disease prevention program at the Engativa Hospital ‐ Bogota. Gestational age was calculated using the last menstrual period and ultrasound measurements in the first trimester. Controls were healthy pregnancies (*n* = 46) and cases were pregnant women diagnosed with mild pre‐eclampsia in the same cohort study (*n* = 20). In this cohort study, the prevalence of severe pre‐eclampsia was low and for this reason, only women diagnosed with mild pre‐eclampsia were included in the study. In addition, twenty (*n* = 20) eumenorrheic women were included in the study during the follicular and luteal phases of the menstrual cycle. Systolic (SBP) and diastolic blood pressure (DBP), anthropometric measures, biochemical and hormonal determinations and a complete medical record history were obtained at each prenatal and postpartum visit.

The diagnosis of pre‐eclampsia was based on ACOG guidelines [SBP ≥ 140 mmHg and/or DBP ≥ 90 mmHg on two occasions taken separated by a 4–6 h period, and proteinuria ≥300 mg/24 h or ≥2 + dipstick].^40^, ^41^ Mild pre‐eclampsia were diagnosed with SBP between 140–159 mmHg or DBP measures between 90–109 mmHg, non‐elevated liver enzymes, absence of renal insufficiency, pulmonary oedema, cyanosis, new‐onset headaches or visual disturbances, and/or right upper quadrant or epigastric pain.^42^

The study included pregnant women who attended routine prenatal visits in the outpatient clinic of the Engativá Hospital of Bogotá, Colombia, between weeks 11 and 13 of gestation, who agreed to participate in the study and signed the informed consent. Nulliparous and multiparous pregnant women with a single pregnancy, who were undergoing a normal pregnancy and who could attend full follow‐up during pregnancy until delivery, and control at 3 months postpartum were included. In addition, the exclusion criteria were pre‐pregnancy hypertension and diabetes mellitus, obesity, autoimmune and metabolic disorders, thyroid disease, liver and renal diseases, acute and chronic infections, diseases of the haematopoietic system, as well as women who were taken medication that affected metabolism.

### 2.3 Biochemical analysis

Biochemical and hormonal analyses in pregnant women were performed in the morning (07:00–08:00 h) after an overnight fast (10:00–12:00 h). Blood samples were drawn through the antecubital vein into 10‐mL plastic BD – Vacutainer® tubes. Serum levels of glucose, triglyceride (TG), total cholesterol (TC), high‐density lipoprotein cholesterol (HDL‐c) and low‐density lipoprotein cholesterol (LDL‐c) were determined by enzymatic methods (Labkit Kits). Additionally, serum levels of insulin were determined by automated chemiluminescence Immunoassay Analyser (Roche), while, serum levels of C‐reactive protein (CRP) were determined by the enzyme‐linked immunosorbent assay. The Homeostasis Model Assessment of Insulin Resistance Index (HOMA‐IR) was determined as described elsewhere.^43^ Serum levels of progesterone were determined in eumenorrheic women by immunoassay (Roche Elecsys 1010 Immunoanalyzer) during the follicular and luteal phases of the menstrual cycle.

Serum levels of human leptin (KAC2281 – Invitrogen) and sOB‐r (DOBR00 – R&D Systems) were analysed by ELISA as described by the manufacturer. Human assay ranges for leptin were 15.6–1000 pg/mL, sensitivity was <3.5 pg/mL and the intra‐ and interassay coefficients of variation (CV) were 4.6% and 3.6%. On the other hand, for human sOB‐r, the analytical sensitivity was <0.128 ng/mL, the assay ranges were 0.3–20 ng/mL and the intra‐ and interassay coefficients of variation (CV) were 5.5% and 5.5%. The ratio between leptin and sOB‐R levels (FLI) was determined as described elsewhere.^26^, ^27^, ^28^

### 2.4 Statistical analyses

For statistical analysis, data are expressed as means ± standard deviation (SD). The Student's *t* test was used to compare the means between two groups. Variables with non‐normal distribution were evaluated with the Mann–Whitney test. For repeated measures, the longitudinal data were compared using analysis of variance (anova). For the linear regression analysis, serum levels of leptin, sOB‐R and FLI were logarithmically transformed. During each trimester of pregnancy, Pearson's correlation coefficient was determined between serum levels of leptin or FLI with different study variables. To assess the predictive value of leptin and FLI for pre‐eclampsia, the receiver operating characteristic (ROC) curve was generated during the first and second trimesters of pregnancy. To calculate the odds ratios (ORs), univariate and multivariate logistic regression analyses were used to predict the risk of mild pre‐eclampsia outcome and its association with FLI, serum leptin, HOMA‐IR and SBP, at the first and second trimesters of pregnancy. A *p* value of <0.05 was considered statistically significant in all analyses. We used STATA 15 ‐ IC® version for statistical analyses.

## 3 RESULTS

### 3.1 General characteristics of pregnant women

Table 1 and Table 2 summarize the general characteristics of pregnant and eumenorrheic women. Table S1 compares different characteristics between pre‐eclamptic and healthy pregnancies. Obese patients, with DM and with pre‐pregnancy chronic diseases were excluded from the present study. Results of the current study were consistent with different longitudinal studies conducted in healthy and pre‐eclamptic pregnant women, where systolic BP, medium BP, HDL‐c, insulin levels and HOMA‐IR are statistically significantly different between these groups of women during pregnancy (Table S1).^44^, ^45^, ^46^ Additionally, results show that serum leptin levels, sOB‐R and FLI were significantly different between healthy and mild pre‐eclamptic women in the second and third trimesters of pregnancy (*p* < 0.05); meanwhile, no significant differences were found in the first trimester of pregnancy (*p* > 0.05; Table S1).

**TABLE 1:** General characteristics of healthy pregnant and eumenorrheic women.

Variables	Eumenorrheic women (*n* = 20)	Healthy pregnant women (*n* = 46)			Postpartum (*n* = 20)
		1st trimester	2nd trimester	3rd trimester	
Age (years)	22.3 ± 3.8 19–25	25.1 ± 6.7 19–31	**–**	**–**	**–**
Gestational age (weeks)	**–**	12.1 ± 0.6 11.5–12.5	24.5 ± 0.7 24.1–24.6	34.8 ± 1.0 34.2–35.4	**–**
BMI (kg/m^2^)	21.3 ± 1.8 19.9–22.9	22.7 ± 2.3 20.8–23.8	24.6 ± 2.4 22.7–25.9	26.5 ± 2.6 24.4–27.9	23.1 ± 2.5 21–24.4
SBP (mmHg)	106.9 ± 9.7 99–115	93.4 ± 7.5 90–100	90.9 ± 9.1 82–100	96.1 ± 8.5 90–102	105.7 ± 24.2 108–116
DBP (mmHg)	69 ± 5.9 65–75	60.6 ± 6.1 58–62	59.5 ± 6.4 58–60	62.1 ± 8.0 58–64	68.1 ± 4.9 65–70
MBP (mmHg)	81.6 ± 6.3 76.7–87.3	71.5 ± 5.8 69.3–74	70.0 ± 6.0 66.7–73.3	73.4 ± 7.5 69.3–78	80.6 ± 7.7 78.3–84
Glucose (mg/dL)	82.2 ± 7.5 78–86	78.9 ± 5.9 74–83	74.5 ± 5.3 69–79	74.2 ± 5.7 71–77	81.4 ± 5.9 77–84
Insulin (μUI/mL)	9.1 ± 5.7 4.5–14.1	9.6 ± 4.3 5.9–11.7	11.3 ± 4.3 8.5–14.3	12.00 ± 5.2 7.7–16.7	6.6 ± 3.8 3.9–9
HOMA Index	1.7 ± 1.3 0.8–2.4	1.9 ± 0.9 1.8–2.2	2.1 ± 0.9 1.5–2.6	2.2 ± 1.0 1.4–3.1	1.3 ± 0.8 0.7–1.9
Total cholesterol (mg/dL)	157.3 ± 27.3 129–178	166.7 ± 31.6 145–190	221.4 ± 39.3 190–255	251. 4 ± 50.6 219–287	158.7 ± 28.3 140–181
HDL (mg/dL)	47.8 ± 8.7 43–52	58.6 ± 9.9 51–65	70.3 ± 12.4 62–78	66.5 ± 11.3 64–74	45.9 ± 10.3 44–53
LDL (mg/dL)	109.5 ± 27.2 90–127	122.5 ± 34.4 94–148	146.2 ± 47.1 109–177	162 ± 44.8 134–190	94.7 ± 29.7 74–113
VLDL (mg/dL)	15.3 ± 4.7 12–19	22.4 ± 8.1 16–27	37.4 ± 12.4 28–44	49.7 ± 15.1 41–58	18.3 ± 10.7 12–23
Triglycerides (mg/dL)	76.1 ± 23.4 59–63	112.2 ± 40.3 81–133	187.0 ± 62.0 140–221	248.4 ± 75.8 205–291	92 ± 53.7 59–116
C‐reactive protein (mg/L)	1.6 ± 1.5 0.6–1.6	5.4 ± 2.7 3.7–7.4	4.8 ± 2.4 3.1–6.6	5.4 ± 3.3 2.6–8.4	3.5 ± 3.9 1.3–4.3
Progesterone					
Follicular	0.5 ± 0.2 0.3–0.7	**–**	**–**	**–**	**–**
Luteal	10.8 ± 5.5 4.6–15.7	**–**	**–**	**–**	**–**
Leptin (ng/mL)		22.8 ± 9.3 16.4–30.1	34.4 ± 18.2 22.0–46.7	38.2 ± 19.5 21.7–52.7	16.0 ± 4.9 11.6–20.2
Follicular	16.5 ± 6.6 13.8–17.3	–	–	–	–
Luteal	22.9 ± 6.4 19.2–27.3	–	–	–	–
sOB‐R (ng/mL)		32.4 ± 7.5 28.1–36.6	43.7 ± 9.3 37.9–49.2	45.0 ± 10.8 35.7–53.3	26.7 ± 3.5 23.6–30.0
Follicular	20.9 ± 2.1 19.9–22.7	–	–	–	–
Luteal	21.6 ± 2.6 18.9–23.5	–	–	–	–
Free Leptin Index		7.8 ± 4.9 4.6–10.3	8.6 ± 6.4 4.4–10.8	9.4 ± 5.9 4.2–11.9	6 ± 1.6 4.9–7.7
Follicular	7.9 ± 2.6 6.2–8.5	–	–	–	–
Luteal	10.9 ± 5.6 7.9–13.6	–	–	–	–

Abbreviations: BMI, Body mass index; DBP, Diastolic blood pressure (mmHg); FLI, Free Leptin Index (Leptin/sOB‐R); Fo, follicular; HDL‐c, High‐Density Lipoprotein Cholesterol; Lu, luteal; MBP, Medium blood pressure (mmHg); SBP, Systolic blood pressure (mmHg); sOB‐R, soluble leptin receptor; VLDL, Very Low‐Density Lipoprotein.

**TABLE 2:** General characteristics of pre‐eclamptic pregnancies.

Variables	Pre‐eclamptic pregnancies (*n* = 20)		
	First trimester	Second trimester	Third trimester
Age (years)	22.4 ± 6.6 18.0–26.5	–	–
Gestational age (weeks)	12.2 ± 0.7 11.5–12.6	24.5 ± 0.6 24.1–24.5	35 ± 0.9 34.2–35.6
BMI, kg/m^2^	24.0 ± 2.8 21.8–25.3	26.4 ± 2.8 24.4–28.1	29.4 ± 2.73 27.7–30.6
SBP (mmHg)	104 ± 7.6 99–110	104.2 ± 8.6 100–110	108.6 ± 12.5 100–115
DBP (mmHg)	65.7 ± 7.6	65.4 ± 7.5	65.3 ± 6.6
	60–70	60–70	60–70
MBP (mmHg)	78.5 ± 7.1 73–82.7	78.3 ± 6.9 74–83.4	79.7 ± 9.2 74.5–81.7
Serum glucose (mg/dL)	80.5 ± 6.8 75.3–84	77.1 ± 7.7 70–83	74.7 ± 9.3 69.5–78
Serum Insulin (μUI/mL)	17.3 ± 23.8 9.8–13.9	15.3 ± 4.5 11.2–18.2	15.3 ± 6.6 11.5–18.6
HOMA Index	3.7 ± 5.9 1.8–2.9	2.9 ± 0.9 2.2–3.6	2.9 ± 1.4 2.1–3.5
Total Cholesterol (mg/dL)	171.1 ± 33.0 159.5–191.5	220.7 ± 44.4 189–244	235.8 ± 48.5 207.5–258.5
HDL (mg/dL)	52.3 ± 12.1 44–58.5	63.7 ± 14.8 51.5–74	57.7 ± 17.6 51–63.5
LDL (mg/dL)	122.7 ± 38.9 91.5–143	152.9 ± 58.0 113–174.5	157.9 ± 68.3 110–198.5
VLDL (mg/dL)	23.1 ± 9.5 16–27	35.9 ± 14.9 26.5–42	49.9 ± 19.3 33.5–64
Triglycerides (mg/dL)	115.2 ± 47.3 78–134.5	179.5 ± 73.9 133–208.5	258.9 ± 85.4 187–321
C‐reactive protein (mg/L)	6.7 ± 3.8 1.0–13.7	7.4 ± 3.0 5.3–10	7.1 ± 3.5 5.2–8.7
Leptin (ng/mL)	24.9 ± 9.9 17.0–31.8	47.1 ± 25.1 29.3–61.2	63.0 ± 30.9 32.2–83.3
sOB‐R (ng/mL)	32.1 ± 7.0 25.7–37.9	37.5 ± 6.3 32.9–43.8	37.0 ± 7.7 31.0–42.9
Free Leptin Index (FLI)	8.7 ± 5.0 4.6–12.4	13.5 ± 8.8 7.4–19.5	18.1 ± 10.4 7.7–25.7

Abbreviations: BMI, Body mass index; DBP, Diastolic blood pressure (mmHg); FLI, Free Leptin Index (Leptin/sOB‐R); HDL‐C, High‐density lipoprotein cholesterol; MBP, Medium blood pressure (mmHg); SBP, Systolic blood pressure (mmHg); sOB‐R, soluble leptin receptor; VLDL, Very low‐density lipoprotein.

On the other hand, gestational age at delivery was significantly different between healthy (39.2 weeks) compared with pre‐eclamptic (37.6 weeks) pregnant women (*p* < 0.05). Additionally, there were no significant differences (*p* > 0.05) in the Apgar score between newborns of healthy and pre‐eclamptic pregnant women in the first (9.0 vs 8.7, respectively) and fifth (9.1 vs 9.3, respectively) minutes. We found a significant reduction in neonatal birth weight in pre‐eclamptic women (2762 ± 577 g) in comparison to healthy pregnant women (3099 ± 305 g) (*p* < 0.05). Additionally, we found that 46.4% of neonates of the pre‐eclamptic mothers' group were admitted to UCI because of low weight or respiratory distress. Finally, in this study, the measurement of uterine artery pulsatility index in the first trimester did not have any statistical difference (*p* > 0.05) between healthy (PI = 1.7) and pre‐eclamptic (PI = 1.9) pregnant women.

### 3.2 Serum levels of leptin during pregnancy

**FIGURE 1:**
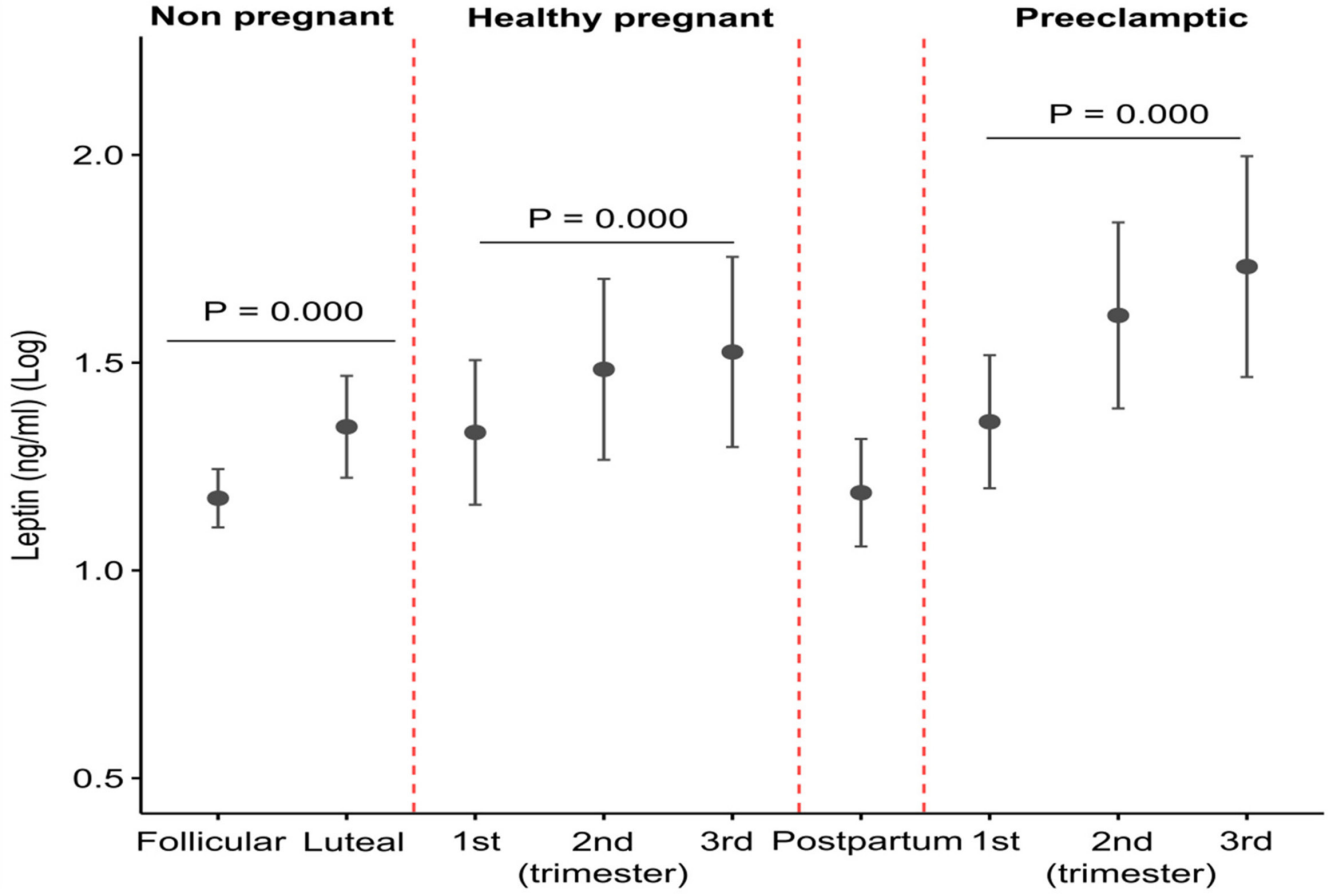
Serum levels of leptin in eumenorrheic women during the follicular and luteal phases of the menstrual cycle and pregnancy in healthy and pre‐eclamptic pregnancies. A *p* < 0.05 indicates a statistically significant difference.

Serum levels of leptin significantly rise in the luteal phase compared to the follicular phase of the menstrual cycle in eumenorrheic women (*p* < 0.01) (Figure 1, Table 1 and Table S2). In healthy pregnancies, serum levels of leptin increased significantly during gestation (*p* < 0.01) (Figure 1, Tables S2 and S3). After delivery, serum levels of leptin markedly dropped (Figure 1 and Table S2).

**FIGURE 2:**
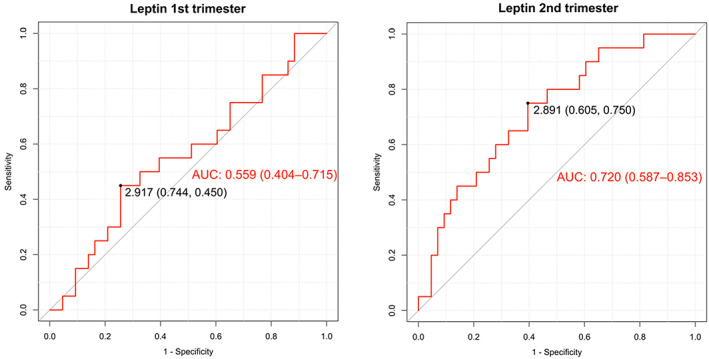
Receiver operating characteristic (ROC) curves for leptin to predict mild pre‐eclampsia during the first and second trimesters of pregnancy. The optimal cut‐off points of serum levels of leptin at each trimester for predicting mild pre‐eclampsia are shown. AUC: area under the curve (CI 95%).

In pre‐eclamptic pregnancies, serum levels of leptin were significantly elevated during pregnancy (*p* < 0.01) (Figure 1, Table 2 and Table S3). A statistically significant difference was found in serum levels of leptin in pre‐eclamptic women compared with healthy pregnant women in second (*p* < 0.05) and third (*p* < 0.01) trimesters of pregnancy (Figure 1, Tables S1 and S4). Pearson's correlation coefficient between serum levels of leptin and study variables in pregnant women during each trimester of pregnancy is shown in Table S9. The ROC curves for leptin as a predictor of mild pre‐eclampsia in the first [AUC = 0.559; 95%CI: 0.404–0.715] and second [AUC = 0.720; 95%CI: 0.587–0.853] trimesters of pregnancy are presented in Figure 2.

### 3.3 Serum levels of sOB‐R in pregnant and eumenorrheic women

**FIGURE 3:**
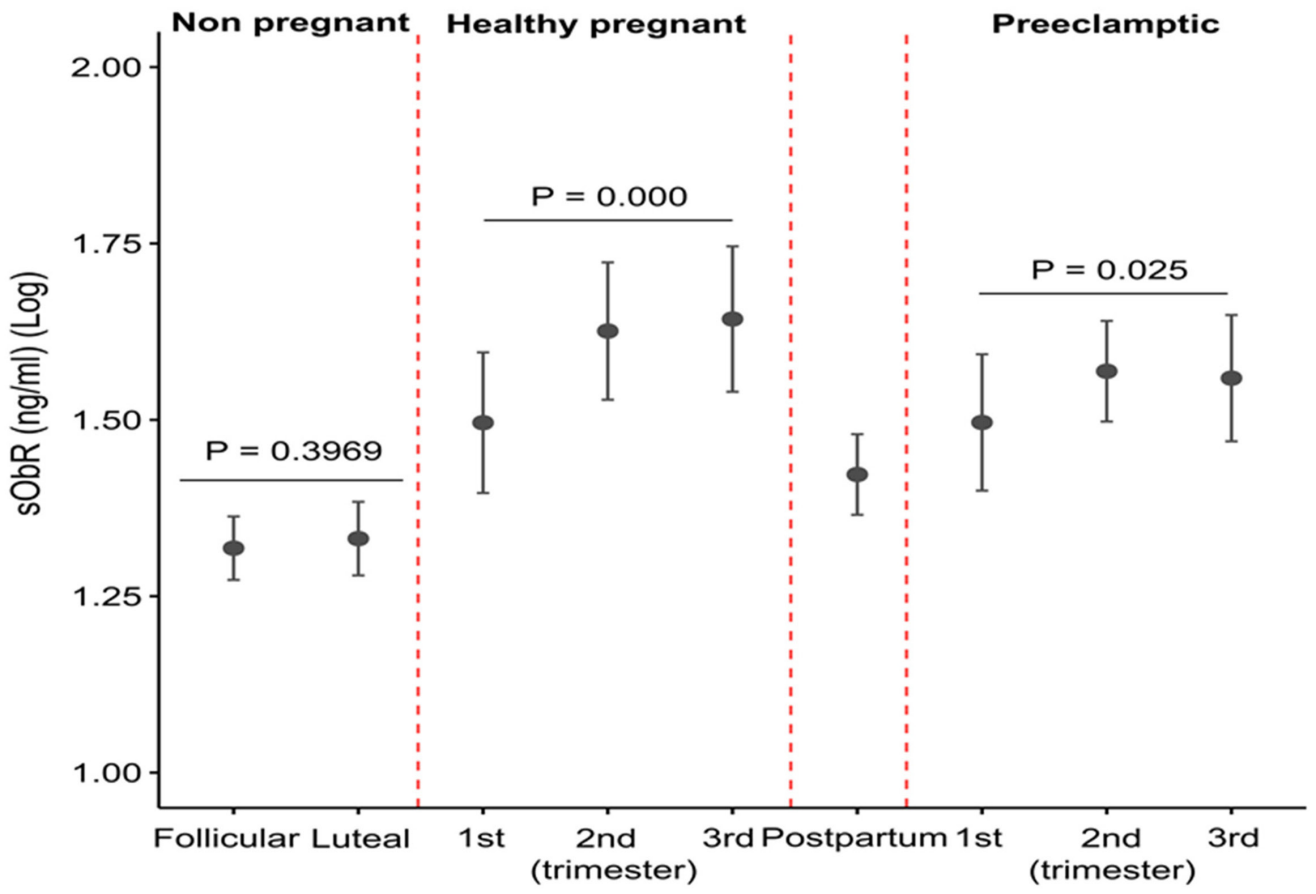
Serum levels of sOB‐R in eumenorrheic women during the follicular and mid‐luteal phases of the menstrual cycle and pregnancy and postpartum in healthy and pre‐eclamptic pregnancies. A *p* < 0.05 indicates a statistically significant difference.

Serum levels of sOB‐R were not statistically different between follicular and luteal phases of the menstrual cycle in eumenorrheic women (*p* > 0.05) (Figure 3 and Table S5). In healthy pregnant women, a significant increase was observed in serum sOB‐R concentrations from the first to third trimesters of pregnancy (*p* < 0.01) and there were no statistically significant differences between the second and third trimesters (*p* > 0.05) (Figure 3, Tables S3 and S5). Serum sOB‐R levels were significantly decreased after delivery (Figure 3 and Table S5).

Circulating sOB‐R increases significantly during pregnancy in pre‐eclamptic women (*p* < 0.05) (Figure 3, Table S3). In addition, in healthy pregnant compared with mild pre‐eclamptic women, serum sOB‐R levels were significantly higher during the second (*p* < 0.01) and third (*p* < 0.01) trimesters of pregnancy (Figure 3 and Table S6).

### 3.4 Free leptin index during pregnancy

**FIGURE 4:**
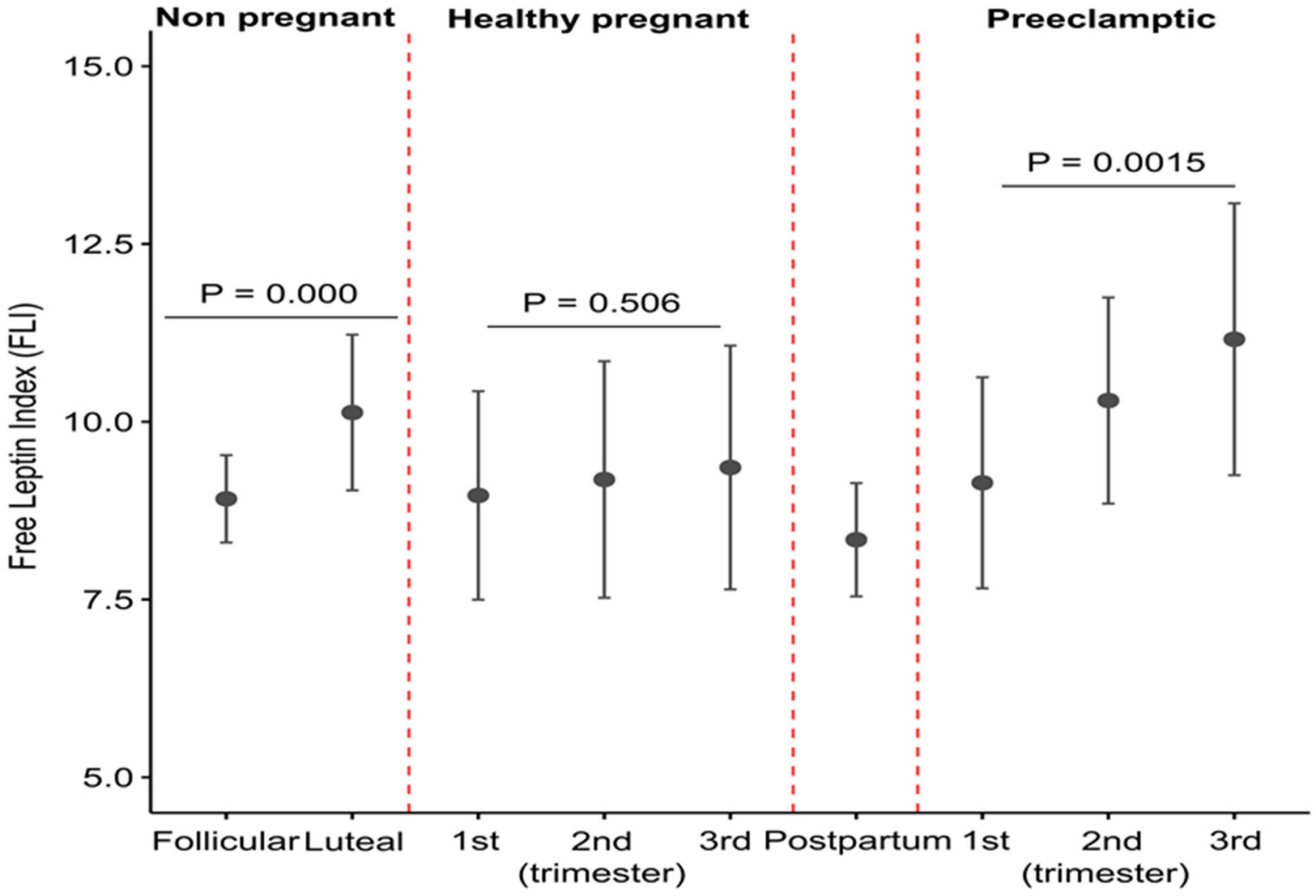
Free Leptin Index (FLI) in eumenorrheic women during the early follicular and luteal phases of the menstrual cycle and pregnancy in healthy and pre‐eclamptic pregnancies. A *p* < 0.05 indicates a statistically significant difference.

FLI was lower in the follicular compared with the luteal phase of the menstrual cycle in eumenorrheic women (Figure 4 and Table S7) (*p* < 0.01). FLI were not significantly different at any trimester of pregnancy in healthy pregnant women (*p* > 0.05) (Figure 4, Tables S3 and S7). Conversely, in pre‐eclamptic pregnant women, FLI increased significantly from the first to third trimesters of pregnancy (Figure 4 and Table S3) (*p* < 0.01). FLI was significantly elevated during the second (*p* < 0.01) and third trimesters (*p* < 0.01) of gestation in pre‐eclamptic compared to healthy pregnant women (Figure 4 and Table S8).

Pearson's correlation coefficient between FLI and different study variables in pregnant women during the first trimester, second trimester and third trimester of pregnancy are shown in Table S10. Furthermore, correlation analysis was made between the FLI and the BMI, and it was found that there was no statistically significant correlation between these two variables in any of the three trimesters of pregnancy (Table S10). Additionally, findings in the current study indicate that there are statistically significant correlations between serum leptin levels and FLI with systolic BP in the second trimester of pregnancy and with serum insulin levels and HOMA‐IR index at each trimester of pregnancy (Table S10), as described elsewhere.^44^, ^45^, ^46^

**FIGURE 5:**
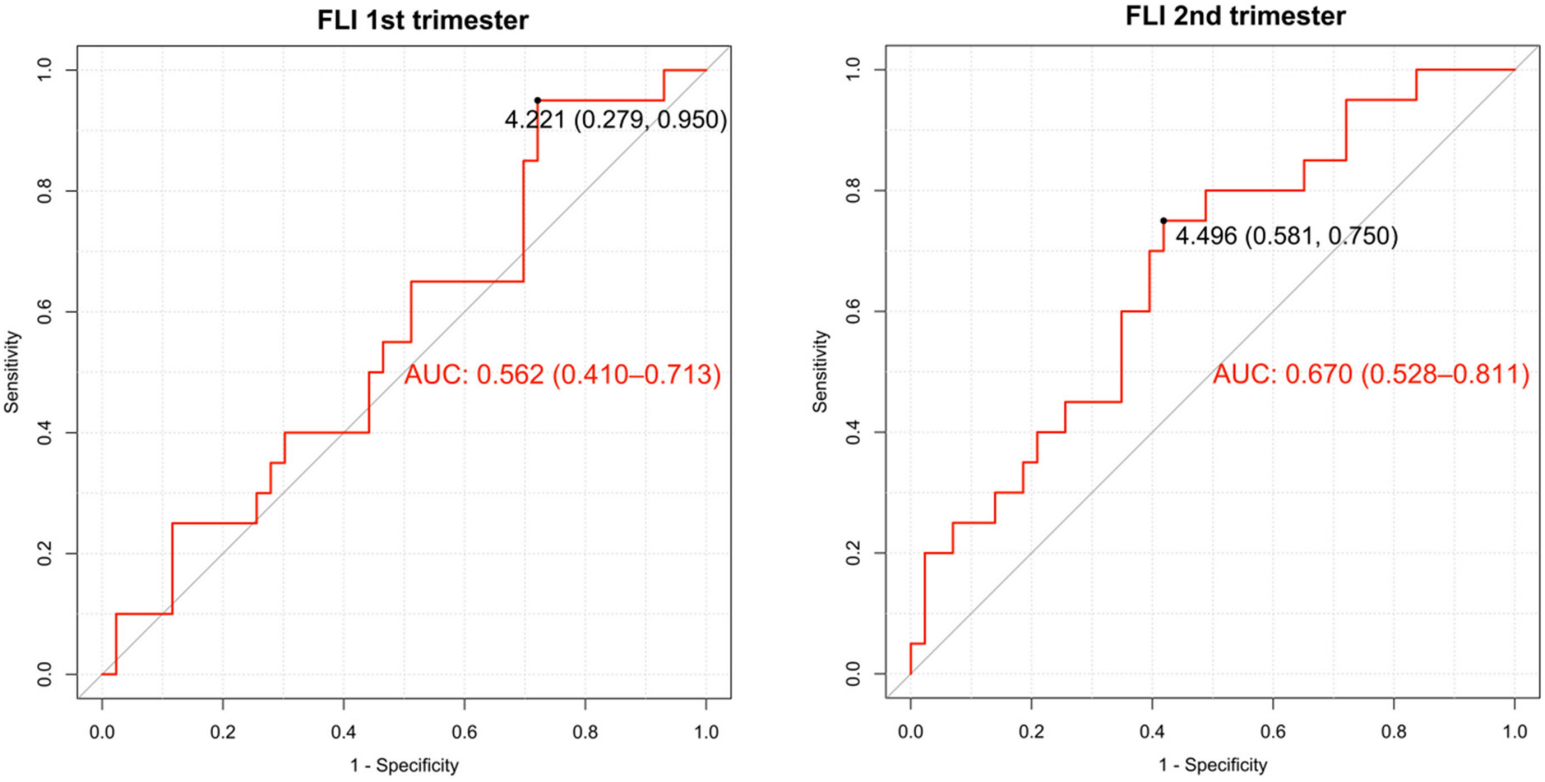
Receiver operating characteristic (ROC) curves for FLI to predict mild pre‐eclampsia during the first and second trimesters of pregnancy. The optimal cut‐off points for FLI at each trimester for predicting mild pre‐eclampsia are shown. AUC: area under the curve (CI 95%).

The ROC curves for FLI as a predictor of mild pre‐eclampsia in the first [AUC = 0.562: 95% CI: 0.410–0.713] and second [AUC = 0.670; 95% CI: 0.528–0.810] trimester of pregnancy are presented in Figure 5.

### 3.5 Univariate and multivariate logistic regression analyses

To predict mild pre‐eclampsia, the univariate and multivariate logistic regression models were evaluated using the variables FLI, serum leptin, HOMA‐IR and SBP (Tables S11 and S12). During the first trimester, FLI, HOMA‐IR and serum leptin levels variables were not associated with mild pre‐eclampsia, while SBP was an accurate predictor of the outcome of mild pre‐eclampsia with a good discrimination capacity in the univariate and multivariate logistic regression analyses (OR: 1.20 [95% CI 1.09–1.31], *p* < 0.05) | (ORa: 1.19 [95% CI 1.08–1.31], *p* < 0.05) (Tables S11.1 and S11.2).

During the second trimester of pregnancy, leptin levels (OR: 1.02 [95% CI 1.00–1.05], *p* < 0.05), FLI (OR: 1.10 [95% CI 1.01–1.18], *p* < 0.05) and HOMA‐IR (OR: 2.80 [95% CI 1.40–5.60], *p* < 0.05) were related to a high probability to predict mild pre‐eclampsia in the univariate logistic regression analysis. Additionally, HOMA‐IR were a strong predictor in the multivariate analysis logistic regression (ORa: 3.38 [95%CI 1.12–10.25], *p* < 0.05). Furthermore, the univariate (OR: 1.20 [95%CI 1.09–1.31], *p* < 0.05) and multivariate (ORa: 1.20 [95%CI 1.08–1.31], *p* < 0.05) logistic regression analysis during the second trimester of pregnancy, shown that SBP was an accurate predictor of mild pre‐eclampsia in the last trimester (Tables S12.1 and S12.2).

## 4 DISCUSSION

In the present study, we demonstrated that FLI is significantly elevated at the second and third trimesters of pregnancy in mild pre‐eclamptic compared with healthy pregnancies. This significant increase in FLI occurs particularly in the second and third trimesters of pregnancy because of a significant increase in circulating levels of leptin with no changes in the serum sOB‐R concentrations over course of pregnancy in pre‐eclamptic women. It is important to highlight that FLI was significantly higher in the luteal phase compared with the follicular phase of the menstrual cycle, possibly in response to the higher serum leptin concentrations and higher progesterone levels in the luteal phase. Additionally, these results are consistent with the previous findings reported by Andersson‐Hall et al. and Donghong Lu et al. in a longitudinal prospective cohort and a cross‐sectional study in healthy pregnant women and women with mild and severe pre‐eclampsia respectively.^13^, ^37^

As it is known, pre‐eclampsia is a multisystem hypertensive disorder during pregnancy and one of the major causes of maternal and fetal morbidity and mortality worldwide.^47^ This disorder has been associated with endothelial dysfunction, coagulopathies, imbalance between pro‐angiogenic and anti‐angiogenic factors, acute kidney injury, oedema, cardiovascular diseases, systemic inflammatory response and oxidative stress.^48^ Furthermore, pre‐eclampsia has been associated with a dysregulated secretion profile of maternal and placental circulating factors, including growth factors, hormones and some adipokines such as leptin.^49^ Leptin is an adipokine that crosses the blood–brain barrier through a saturable transport system to reach the hypothalamus and activate the sympathetic nervous system and which may lead to hypertension.^50^, ^51^, ^52^ Previous studies have shown that high‐circulating leptin levels are present in animals and humans with hypertension.^50^, ^51^, ^52^, ^53^, ^54^

Ole‐Petter R. Hamnvik et al. performed a cross‐sectional Cyprus Metabolism Study and observed a significant inverse correlation between blood pressure and serum sOB‐R levels, meanwhile, it had a direct correlation with leptin.^55^ In a consistent manner, we found that in our study, higher leptin levels, lower sOB‐R levels and higher blood pressure in women with pre‐eclampsia during the second and third trimesters of gestation. Additionally, we found circulating levels of leptin significantly elevated during the second and third trimesters of gestation in both healthy and pre‐eclamptic women. However, this increase is remarkable in pre‐eclamptic pregnancies, suggesting that high leptin levels could contribute to the pathophysiology and underlying mechanisms of hypertensive disorders during pregnancy. These results are consistent with findings reported in patients with primary hypertension and support the hypothesis that there is a strong relationship between hypertension, leptin and sOB‐R.^56^ Our results emphasize and extend the previous reports showing that hyperleptinemia may precede and contribute to the development of hypertension, rather than being a major cause of it. Hyperleptinemia occurs in pre‐eclamptic pregnant women from the second trimester of gestation.^34^ Also, FLI was more strongly related to adverse clinical outcomes and associated with masked hypertension than leptin or sOB‐R alone, suggesting that leptin and its receptor acting conjointly may be involved in the hypertensive disorders of pregnancy. Therefore, the FLI profile seen in pre‐eclamptic pregnant women might be useful for early prediction of pre‐eclampsia in the early second trimester of pregnancy, and therefore might allow timely diagnosis and management with anti‐hypertensive therapy. Thus, ROC curves were constructed to determine the ability of serum leptin levels and FLI to predict mild pre‐eclampsia in the first and second trimesters of pregnancy, since at that time great decisions may be made regarding the treatment and prevention of complications. In this longitudinal study, we showed that pre‐eclampsia is associated with hyperleptinemia, low levels of sOB‐R and increased FLI. Thus, it is possible in pre‐eclamptic pregnant women that elevated FLI and high free leptin levels might favour leptin transport through the blood–brain barrier and the subsequent renal sympathetic hyperactivation via induction of its own receptor in the paraventricular nucleus and lead to an increase in blood pressure.^57^

Recently, to provide an overview of the accurate prediction of pre‐eclampsia risk from changes in the maternal variation profile in some adipokines throughout pregnancy, Georgios Daskalakis et al. analysed the data of 163 studies conducted in 23.482 women.^58^ Thus, in 91/163 studies, it was demonstrated that serum leptin levels rise significantly at each trimester of pregnancy in pre‐eclamptic women compared to healthy pregnant women, independent of the onset and severity of the illness and in this way, high leptin levels have been considered the best predictor hormone biomarker of pre‐eclampsia and hypertensive disorders of pregnancy compared to other adipocytokines.^58^ Additionally, it is important to highlight that different studies have shown that leptin levels are significantly higher in women diagnosed with severe pre‐eclampsia compared to mild pre‐eclampsia.^58^ Thus, elevated leptin levels in the first trimester of pregnancy in combination with different risk factors such as hypertension, obesity and diabetes mellitus is currently the main adipocytokine to accurately predict severe and early onset pre‐eclampsia. On the other hand, different adipokine profiles have been determined in pre‐eclamptic women, particularly through cross‐sectional studies, which in combination with leptin levels, could be a strong and accurate predictor of pre‐eclampsia and hypertensive disorders of pregnancy around the mid‐pregnancy for mild pre‐eclampsia.^58^ In this way, longitudinal studies of FLI during pregnancy and in combination with chronic diseases, such as diabetes, obesity and hypertensive illness, could contribute as an accurate predictor biomarker of pre‐eclampsia and hypertensive disorders compared to other adipocytokines.

Finally, the present longitudinal study was conducted taking into count the prevalence of hypertensive pregnancy‐related complications of 5% in our population, where mild pre‐eclampsia was one of the main maternal adverse outcomes with few cases of diagnosis of severe pre‐eclampsia/eclampsia. Thus, in this prospective study of 465 women, only cases of mild pre‐eclampsia occurred, there were no cases of severe pre‐eclampsia or eclampsia, which is a limitation of this study. Therefore, longitudinal studies should be developed that include severe pre‐eclamptic/eclamptic women to determine the profile of the free Leptin Index (FLI) during pregnancy because it could be a possible biomarker for early prediction of pregnancy‐related hypertensive disorders and could contribute to appropriate management and prevention of pregnancy complications.

## 5 CONCLUSIONS

This longitudinal study during pregnancy, and 3 months postpartum in mild pre‐eclamptic women show that circulating levels of leptin and sOB‐R concentration might play a critical role in the pathophysiology of mild pre‐eclampsia. Additionally, our results demonstrated a statistically significant increase of FLI in women with mild pre‐eclampsia compared with normotensive pregnant women mainly caused by the high‐serum levels of leptin as pregnancy progressed. In this way, during the first and second trimesters of pregnancy, SBP was considered the strongest independent predictor for mild pre‐eclampsia, which is also a criterion for the diagnosis of this hypertensive disorder. However, the association of the biochemical biomarkers during the second trimester suggests a relationship between insulin resistances, serum leptin and FLI with mild pre‐eclampsia.

## AUTHOR CONTRIBUTIONS

**Maria Fernanda Garcés:** Investigation (equal); methodology (equal). **Julieth Daniela Buell‐Acosta:** Formal analysis (equal); methodology (equal). **Haiver Antonio Rodriguez‐Navarro:** Data curation (equal); methodology (equal). **María Carolina Páez‐Leal:** Investigation (equal); methodology (equal); writing – original draft (equal). **Luis Miguel Maldonado‐Acosta:** Writing – original draft (equal). **Jhon Jairo Peralta‐Franco:** Investigation (equal); methodology (equal). **Álvaro Javier Burgos‐Cardenas:** Conceptualization (equal); data curation (equal); investigation (equal); methodology (equal). **Edith Angel‐Muller:** Writing – original draft (equal). **Arturo José Parada‐Baños:** Writing – original draft (equal). **Mario Orlando Parra‐Pineda:** Writing – original draft (equal). **Javier Eslava‐Schmalbach:** Data curation (equal); writing – original draft (equal). **Camilo Andres Escobar‐Sarmiento:** Conceptualization (equal); data curation (equal); formal analysis (equal). **Ezequiel Lacunza:** Data curation (equal); formal analysis (equal); investigation (equal); methodology (equal). **Sofia Alexandra Caminos‐Cepeda:** Data curation (equal); writing – original draft (equal). **Justo P. Castaño:** Writing – original draft (equal); writing – review and editing (equal). **Rubén Nogueiras:** Writing – original draft (equal). **Carlos Dieguez:** Writing – original draft (equal). **Ariel Iván Ruíz‐Parra:** Supervision (equal); writing – original draft (equal). **Jorge Eduardo Caminos:** Writing – original draft (equal); writing – review and editing (equal).

## FUNDING INFORMATION

This study was supported by government grants to the Universidad Nacional de Colombia (DIEB) and School of Medicine (código Hermes: 41439) and Ministerio de Ciencia, Tecnología e Innovación (Minciencias) (CD: 202010012913‐2019‐INV‐Colciencias). Additionally, this work was supported by grants from FEDER/Ministerio de Ciencia, Innovación y Universidades‐Agencia Estatal de Investigación (CD: BFU2017‐87721; RN: RTI2018‐099413‐B‐I00); Xunta de Galicia (RN: 2021‐CP085 and 2020‐PG0157).

## CONFLICT OF INTEREST STATEMENT

The authors report no conflict of interest in this work.

## Supporting information


Table S1

## Data Availability

The requested information is attached in the Supporting Information.
