# Supplementary material for: A longitudinal study of free leptin index in pre‐eclamptic pregnancies
Source: J Cell Mol Med. 2023 Mar 23;27(8):1083–94. doi: 10.1111/jcmm.17707 (PMC10098289; doi:10.1111/jcmm.17707)
Supplement: Supplementary file 1 — Table S1 [file JCMM-27-1083-s001.pdf]

Supplementary Material

A Longitudinal study of Free Leptin Index (FLI) in Preeclamptic Pregnancies

María Fernanda Garcés[1], Julieth Daniela Buell – Acosta[1], Haiver Antonio Rodríguez – Navarro[1], María Carolina Páez – Leal[2], Luis Miguel Maldonado - Acosta[3], Jhon Jairo Peralta - Franco[3], Álvaro Javier Burgos - Cardenas[4], Edith Ángel - Müller[5], Arturo José Parada – Baños[5], Mario Orlando Parra - Pineda[5], Javier Eslava – Schmalbach[6], Camilo Andrés Escobar – Sarmiento[7], Ezequiel Lacunza[8], Sofia Alexandra Caminos – Cepeda[9], Justo P Castaño[10,11], Rubén Nogueiras[11,12], Carlos Dieguez[11,12], Ariel Iván Ruiz – Parra[5] and Jorge Eduardo Caminos[1]

Department of Physiology[1], Department of Public Health[2], Division of Endocrinology - Department of Internal Medicine[3], Department of Internal Medicine[4], Department of Obstetrics and Gynecology[5] and Department of Surgery[6], School of Medicine Universidad Nacional de Colombia, Bogotá 11001, Colombia. Fundación Sueño Vigilia Colombiana - Bogotá 111211, Colombia[7]. Centro de Investigaciones Inmunológicas Básicas y Aplicadas (CINIBA), Facultad de Ciencias Médicas, Universidad Nacional de La Plata, La Plata 1900, Argentina[8]. School of Medicine, Universidad Pompeu Fabra, Barcelona 08002 - Spain[9]. Department of Cell Biology, Physiology, and Immunology, Institute Maimonides for Biomedical Research of Cordoba, Reina Sofia University Hospital, University of

Cordoba, Cordoba 14004, Spain[10]. CIBER Fisiopatología de la Obesidad y Nutrición, Instituto de Salud Carlos III, Madrid 28029 - Spain[11]. Department of Physiology (CIMUS), School of Medicine - Instituto de Investigaciones Sanitarias (IDIS), Universidad de Santiago de Compostela, Santiago de Compostela 15782 - Spain[12]

Correspondence: Jorge Eduardo Caminos, MSc. PhD. Department of Physiology, School of Medicine. Universidad Nacional de Colombia. Carrera 30 No. 45-03, Edificio 471 Piso 4 Oficina 406, Bogotá, 15 Colombia. Tele/fax: 57-1-316-5464.

E-mail: jecaminosp@unal.edu.co

Short running head: Free Leptin Index in preeclamptic pregnancies

Supplementary Table 1: Comparison of baseline variables between preeclamptic women and healthy normotensive pregnant women (log-transformed) during the 1st trimester, 2nd trimester and 3rd trimester of pregnancy. Log transformed values were used for testing differences variables. *P<0.05.

Variables	1st trimester<br>Healthy Vs Preeclamptic P value*	2nd trimester<br>Healthy Vs Preeclamptic P value*	3rd trimester<br>Healthy Vs Preeclamptic P value*
Age (years)	0.1313	–	–
Gestational age (weeks)	0.5009	0.5733	0.7486
BMI (kg/m^2)	0.0508	0.0112	0.0001
SBP (mmHg)	0.0000	0.0000	0.0000
DBP (mmHg)	0.0068	0.0013	0.1050
MBP (mmHg)	0.0001	0.0000	0.0026
Blood glucose (mg/dL)	0.3528	0.1690	0.9234
Insulin (µUI/mL)	0.0090	0.0021	0.0745
HOMA Index	0.0102	0.0015	0.0882
Total cholesterol (mg/dL)	0.6713	0.9136	0.2446
HDL (mg/dL)	0.0203	0.0444	0.0086
LDL (mg/dL)	0.9072	0.6897	0.4324
VLDL (mg/dL)	0.9824	0.4098	0.6932
Triglycerides (mg/dL)	0.3528	0.1690	0.9234
C –Reactive protein	0.3210	0.0029	0.0767

Leptin (ng/mL)	0.2976	0.0179	0.0047
sOB-R (ng/mL)	0.7576	0.0077	0.0026
FLI	0.3824	0.0050	0.0005

The Mann–Whitney U test was used for comparisons of continuous Log transformed values. Abbreviations: BMI, Body mass index; HDL-C, High-Density Lipoprotein Cholesterol; VLDL, Very Low-Density Lipoprotein; SBP, Systolic blood pressure (mmHg); DBP, Diastolic blood pressure (mmHg); MBP, Medium blood pressure (mmHg); sOB-R, soluble leptin receptor; FLI, free leptin index (leptin/sOB-R). A p value of < 0.05 was considered as statistically significant. Log-transformed (log10) values were used. Gray boxes are used to indicate statistical significance between healthy and preeclamptic women results.

Supplementary Table 2: Comparison of serum leptin levels during the three gestational periods and postpartum in healthy pregnant women and healthy non- pregnant women.

Healthy Pregnant women

Healthy Pregnant women	Follicular	Luteal	1st trimester	2nd trimester	3rd trimester	Post-partum
Follicular		0.0000	0.0003	0.0000	0.0000	0.6637
Luteal	0.0000		0.7593	0.0118	0.0020	0.0003
1st trimester	0.0003	0.7593		0.0004	0.0000	0.0014
2nd trimester	0.0000	0.0118	0.0004		0.3718	0.0000
3rd trimester	0.0000	0.0020	0.0000	0.3718		0.0000

Comparison between serum leptin levels during the three gestational periods and postpartum in healthy pregnant women. Statistical differences were evaluated through the Student t-test of independent samples assuming unequal variances. A p value of < 0.05 was considered as statistically significant. Gray boxes are used to indicate statistical significance between groups.

Supplementary Table 3:

Longitudinal changes of baseline variables in preeclamptic women and normotensive pregnant women (log-transformed) across the 1st trimester, 2nd trimester and 3rd trimester of pregnancy. Log transformed values were used for testing differences variables. *P<0.05.

**Variable**	**Healthy Pregnant women 1st, 2nd and 3rd trimester P value***	**Preeclamptic Pregnant women 1st, 2nd and 3rd trimester P value***
BMI (kg/m²)	0.0000	0.0000
SBP (mmHg)	0.0158	0.2922
DBP (mmHg)	0.2355	0.9875
MBP (mmHg)	0.0538	0.7992
Blood glucose (mg/dL)	0.0003	0.0550
Insulin (µUI/mL)	0.0686	0.6733
HOMA Index	0.2896	0.8085
Total cholesterol (mg/dL)	0.0000	0.0000
HDL (mg/dL)	0.0000	0.0720
LDL (mg/dL)	0.0002	0.1250
VLDL (mg/dL)	0.0000	0.0000
Triglycerides (mg/dL)	0.0003	0.0550
C –Reactive protein	0.6729	0.6589
Leptin (ng/mL)	0.0000	0.0000
sOB-R (ng/mL)	0.0000	0.0257
FLI	0.5068	0.0015

* One-Way ANOVA test was used for comparisons of continuous Log transformed values. Abbreviations: BMI, Body mass index; HDL-C, High-Density Lipoprotein Cholesterol; VLDL, Very Low-Density Lipoprotein; SBP, Systolic blood pressure (mmHg); DBP, Diastolic blood pressure (mmHg); MBP, Medium blood pressure (mmHg); sOB-R, soluble leptin receptor; FLI, free leptin index (leptin/sOB-R). A p value of < 0.05 was considered as statistically

significant. Log-transformed (log10) values were used. Gray boxes are used to indicate statistical significance between groups.

Supplementary Table 4: Comparison of serum leptin levels in three gestational periods between healthy pregnant and preeclamptic women.

		Preeclamptic women				
		**Follicular**	**Luteal**	**1st trimester**	**2nd trimester**	**3rd trimester**
**Healthy Pregnant women**	**Follicular**		0.0000	0.0000	0.0000	0.0000
	**Luteal**	0.0000		0.6495	0.0000	0.0000
	**1st trimester**	0.0003	0.7593	0.2976		
	**2nd trimester**	0.0000	0.0118		0.0179	
	**3rd trimester**	0.0000	0.0020			0.0047

Comparison between serum leptin levels in three gestational periods in healthy pregnant women and Preeclamptic women. Statistical differences were evaluated through the Student t-test of independent samples assuming unequal variances. A p value of < 0.05 was considered as statistically significant. Gray boxes are used to indicate statistical significance between groups.

Supplementary Table 5: Comparison of serum soluble leptin receptor (sOB-R) levels in three gestational periods and postpartum in healthy pregnant women and healthy non-pregnant women.

	Healthy Pregnant women					
**Healthy Pregnant women**	**Follicular**	**Luteal**	**1st trimester**	**2nd trimester**	**3rd trimester**	**Post-partum**
**Follicular**		0.3969	0.0000	0.0000	0.0000	0.0000
**Luteal**	0.3969		0.0000	0.0000	0.0000	0.0000
**1st trimester**	0.0000	0.0000		0.0000	0.0000	0.0036
**2nd trimester**	0.0000	0.0000	0.0000		0.3999	0.0000
**3rd trimester**	0.0000	0.0000	0.0000	0.3999		0.0000

Comparison between serum soluble leptin receptor (sOB-R) levels in three gestational periods and postpartum in healthy pregnant women. Statistical differences were evaluated through the Student t-test of independent samples assuming unequal variances. A p value of < 0.05 was considered as statistically significant. Gray boxes are used to indicate statistical significance between groups.

Supplementary Table 6: Comparison of serum soluble leptin receptor (sOB-R) levels between three gestational periods in healthy pregnant and preeclamptic women.

		Preeclamptic women				
		Follicular	Luteal	1st trimester	2nd trimester	3rd trimester
Healthy Pregnant women	Follicular		0.3969	0.0000	0.0000	0.0000
	Luteal	0.3969		0.0000	0.0000	0.0000
	1st trimester	0.0000	0.0000	0.7576		
	2nd trimester	0.0000	0.0000		0.0077	
	3rd trimester	0.0000	0.0000			0.0026

Comparison between serum soluble leptin receptor (sOB-R) levels in three gestational periods in healthy pregnant women and Preeclamptic women. Statistical differences were evaluated through the Student t-test of independent samples assuming unequal variances. A p value of < 0.05 was considered as statistically significant. Gray boxes are used to indicate statistical significance between groups.

Supplementary Table 7: Comparison of free leptin index (FLI) in three gestational periods and postpartum in healthy pregnant women and healthy non- pregnant women.

		Healthy Pregnant women					
		**Follicular**	**Luteal**	**1st trimester**	**2nd trimester**	**3rd trimester**	**Post-partum**
**Healthy Pregnant women**	**Follicular**		0.0004	0.4450	0.6893	0.9544	0.0043
	**Luteal**	0.0004		0.0037	0.0166	0.0488	0.0000
	**1st trimester**	0.4450	0.0037		0.7222	0.4630	0.2296
	**2nd trimester**	0.6893	0.0166	0.7222		0.7081	0.1629
	**3rd trimester**	0.9544	0.0488	0.4630	0.7081		0.1015

Comparison between free leptin index (FLI) in three gestational periods and postpartum in healthy pregnant women. Statistical differences were evaluated through the Student t-test of independent samples assuming unequal variances. A p value of < 0.05 was considered as statistically significant. Gray boxes are used to indicate statistical significance between groups.

Supplementary Table 8: Comparison of free leptin index (FLI) in three gestational periods between healthy pregnant and preeclamptic women.

Healthy Pregnant women		Preeclamptic women				
		Follicular	Luteal	1st trimester	2nd trimester	3rd trimester
	Follicular		0.0004	0.9730	0.0094	0.0437
	Luteal	0.0004		0.0382	0.5128	0.0437
	1st trimester	0.4450	0.0037	0.3824		
	2nd trimester	0.6893	0.0166		0.0050	
	3rd trimester	0.9544	0.0488			0.0005

Comparison of free leptin index (FLI) in three gestational periods in healthy pregnant women and Preeclamptic women. Statistical differences were evaluated through the Student t-test of independent samples assuming unequal variances. A p value of < 0.05 was considered as statistically significant. Gray boxes are used to indicate statistical significance between groups.

Supplementary Table 9. Pearson's correlation coefficient between serum Leptin levels and study variables in pregnant women during the 1st trimester, 2nd trimester and 3rd trimester of pregnancy. A p value of < 0.05 was considered as statistically significant. Log-transformed (log10) values were used.

Variable	Pregnant women 1st trimester		Pregnant women 2nd trimester		Pregnant women 3rd trimester	
	R - value	P-value	R - value	P-value	R - value	P-value
BMI (kg/m^2)	0.0919	0.4929	0.0071	0.9581	0.0644	0.6309
SBP (mmHg)	0.1799	0.1653	0.3506	0.0056	0.1483	0.2541
DBP (mmHg)	0.1128	0.3869	0.1840	0.1557	0.1157	0.3744
MBP (mmHg)	0.2040	0.1117	0.3770	0.0025	0.2173	0.0898
Blood glucose (mg/dL)	0.1412	0.2778	0.1280	0.3254	0.1676	0.1968
Insulin (µUI/mL)	0.3322	0.0089	0.4853	0.0001	0.3381	0.0077
HOMA Index	0.3062	0.0173	0.4776	0.0001	0.3505	0.0056
Total cholesterol (mg/dL)	0.0980	0.4526	0.1879	0.1471	-0.1694	0.1918
HDL (mg/dL)	0.0052	0.9681	0.0542	0.6783	0.0784	0.5483
LDL (mg/dL)	0.1966	0.1290	0.2794	0.0292	-0.2595	0.0434
VLDL (mg/dL)	0.8395	0.0265	-0.2023	0.1179	-0.0083	0.9491
Triglycerides (mg/dL)	0.1412	0.2778	0.1280	0.3254	0.1676	0.1968

C –Reactive protein	0.3470	0.0071	0.1739	0.1802	0.2639	0.0416

Abbreviations: BMI. Body mass index; HDL-C. High-Density Lipoprotein Cholesterol; VLDL. Very Low-Density Lipoprotein; SBP. Systolic blood pressure at blood draw (mmHg); DBP. Diastolic blood pressure (mmHg); MAP. Medium blood pressure (mmHg); sOB-R. Soluble leptin receptor; FLI. free leptin index (leptin/sOB-R). A p value of < 0.05 was considered as statistically significant. Log-transformed (log10) values were used. Gray boxes are used to indicate statistical significance.

Supplementary Table 10. Pearson's correlation coefficient between FLI and study variables in pregnant women during the 1st trimester, 2nd trimester and 3rd trimester of pregnancy. A p value of < 0.05 was considered as statistically significant. Log-transformed (log10) values were used.

Variable	Pregnant women 1st trimester		Pregnant women 2nd trimester		Pregnant women 3rd trimester	
	R - value	P-value	R - value	P-value	R - value	P-value
BMI (kg/m^2)	0.0754	0.5602	0.2546	0.0458	0.0741	0.5672
SBP(mmHg)	0.1584	0.2150	0.4309	0.0004	0.2408	0.0573
DBP (mmHg)	0.0405	0.7546	0.2391	0.0613	0.1988	0.1214
MBP (mmHg)	0.0980	0.4484	0.3058	0.0156	0.2117	0.0986
Blood glucose (mg/dL)	0.1083	0.4023	0.1077	0.4048	0.1302	0.3130
Insulin (μUI/mL)	0.2976	0.0198	0.5264	0.0000	0.3419	0.0065
HOMA Index	0.5232	0.0000	0.6227	0.0000	0.5704	0.0000
Total cholesterol (mg/dL)	0.1172	0.3645	0.1859	0.1480	-0.2226	0.0820
HDL (mg/dL)	0.0521	0.6877	0.0227	0.8609	0.0078	0.9518
LDL (mg/dL)	0.1733	0.1781	0.3361	0.0076	-0.2745	0.0309
VLDL (mg/dL)	0.0084	0.9483	-0.1987	0.1215	-0.0262	0.8397
Triglycerides (mg/dL)	0.1083	0.4023	0.1077	0.4048	0.1302	0.3130

C –Reactive protein	0.3299	0.0100	0.2673	0.0357	0.3442	0.0066

Abbreviations: BMI. Body mass index; HDL-C. High-Density Lipoprotein Cholesterol; VLDL. Very Low-Density Lipoprotein; SBP. Systolic blood pressure (mmHg); DBP. Diastolic blood pressure (mmHg); MBP. Medium blood pressure (mmHg); sOB-R. Soluble leptin receptor; FLI. free leptin index (leptin/sOB-R). A p value of < 0.05 was considered as statistically significant. Log-transformed (log10) values were used. Gray boxes are used to indicate statistical significance.

Supplementary Table 11.1: Univariate logistic regression analysis used to predict the risk of mild preeclampsia outcome using the variables FLI, serum leptin, HOMA-IR and SBP, during the first trimester of pregnancy

Variables	OR (CI 95%)	p value	C-statistic (CI95%)
FLI	1.04 (0.94 - 1.16)	0.3	0.57 (0.42 - 0.73)
SBP	1.19 (1.08 - 1.31)	0.03	0.84(0.76 - 0.94)
HOMA-IR	1.82 (1.00 - 3.39)	0.06	0.69 (0.55 - 0.83)
Leptin	1.02 (0.96 - 1.08)	0.4	0.56 (0.40 - 0.71)

Abbreviations: SBP, Systolic blood pressure (mmHg); FLI, free leptin index (leptin/sOB-R) and Homeostasis Model Assessment of Insulin Resistance Index (HOMA-IR). A p value of < 0.05 was considered as statistically significant.

Supplementary Table 11.2: Multivariate logistic regression analysis used to predict the risk of mild preeclampsia outcome using the variables FLI, HOMA-IR and SBP, during the first trimester of pregnancy

Variables	OR (CI 95%)	p value	C-statistic (CI95%)
FLI	0.95 (0.80 - 1.12)	0.53	0.87 (0.77 - 0.96)
SBP	1.19 (1.08 - 1.31)	<0.001	
HOMA-IR	2.32 (0.87 - 6.15)	0.09	

Variables	OR (CI 95%)	p value	C-statistic (CI95%)
FLI	1.03 (0.91 - 1.17)	0.6	0.87 (0.78 - 0.96)
SBP	1.19 (1.08 - 1.31)	<0.001	

Abbreviations: SBP, Systolic blood pressure (mmHg); FLI, free leptin index (leptin/sOB-R). A p value of < 0.05 was considered as statistically significant.

Supplementary Table 12.1: Univariate logistic regression analysis used to predict the risk of mild preeclampsia outcome using the variables FLI, serum leptin, HOMA-IR and SBP, during the second trimester of pregnancy

Variables	OR (CI 95%)	p value	C-statistic (CI95%)
FLI	1.10 (1.01 - 1.18)	0.02	0.71 (0.58 - 0.85)
SBP	1.20 (1.09 - 1.31)	<0.01	0.85(0.76 - 0.94)
HOMA-IR	2.80 (1.40 - 5.60)	0.03	0.73 (0.60 - 0.87)
Leptin	1.02 (1.00 - 1.05)	0.04	0.67 (0.53 - 0.81)

Abbreviations: SBP, Systolic blood pressure (mmHg); FLI, free leptin index (leptin/sOB-R) and Homeostasis Model Assessment of Insulin Resistance Index (HOMA-IR). A p value of < 0.05 was considered as statistically significant.

Supplementary Table 12.2: Multivariate logistic regression analysis used to predict the risk of mild preeclampsia outcome using the variables FLI, HOMA-IR and SBP, during the second trimester of pregnancy

Variables	OR (CI 95%)	p value	C-statistic (CI95%)
FLI	0.97 (0.86 - 1.08)	0.54	0.9 (0.82 - 0.97)
SBP	1.20 (1.08 - 1.33)	<0.001	
HOMA-IR	3.38 (1.12 - 10.25)	0.03	

Variables	OR (CI 95%)	p value	C-statistic (CI95%)
FLI	1.04 (0.96 - 1.14)	0.33	0.90 (0.82 - 0.97)
SBP	1.19 (1.08 - 1.31)	<0.001	

Abbreviations: SBP, Systolic blood pressure (mmHg); FLI, free leptin index (leptin/sOB-R) and Homeostasis Model Assessment of Insulin Resistance Index (HOMA-IR). A p value of < 0.05 was considered as statistically significant.
